# On the Central Case Methodology in Discrimination Law

**DOI:** 10.1093/ojls/gqab003

**Published:** 2021-02-15

**Authors:** Shreya Atrey

**Keywords:** central case, discrimination law, intersectionality, intersectional discrimination, grounds

## Abstract

The central case methodology captures the features that something must have in order for it to be that thing. As applied to the field of discrimination law, the methodology helps identify both the central cases of discrimination as well as the key features of discrimination law which address such discrimination. Theorists typically conceive central cases of discrimination law from norms prohibiting discrimination on particular grounds, such as race or sex. This gives the impression that discrimination based on more than one ground is not a central case of discrimination or not prohibited by discrimination law. This article challenges that impression. It argues that, although discrimination based on a combination of grounds (‘intersectional discrimination’) is considered no more than a peripheral case in discrimination law, it should actually be considered ‘more than’ a central case itself. This is because it is epistemically able to capture discrimination in a wider sense. With this, the article seeks to orientate the theoretical discourse in discrimination law to the correct application of the central case methodology which necessitates the consideration of intersectional discrimination.

## 1. Introduction

In his influential account of discrimination, John Gardner proclaims that: ‘If you turn people down for jobs because they are women, *or* refuse to rent flats to people because they are black, then you wrong those people by discriminating against them.’^1^ According to him, ‘It is one thing to discriminate against people on the ground of their vanity or their bullying, but surely quite another to discriminate against them on the ground of their race *or* their sex’^2^ or ‘[to] refuse to drink with people of other races *or* who regard a woman’s proper place as being in the home, is not the same as being prejudiced, gullible or superstitious oneself’.^3^ For Gardner, discrimination is wrongful only when people are denied autonomous life choices based on their identities or grounds, which are either immutable (like race or sex) or a fundamental choice of the individual (like religion or sexuality). Grounds such as race, sex, sexual orientation or religion thus make a crucial difference to our understanding of discrimination. Grounds of discrimination matter—in that discrimination is something that is *based on* them. Every example that Gardner invokes of discrimination based on race *or* sex is an example of discrimination based not just on either of them, but necessarily on one of them at a time. Discrimination thus appears to be not only that which is *based on* grounds, but that which is based on *a* ground specifically. Gardner does not bring up any example where several grounds are involved; say, when Black women—who are Black *and* women—are turned down for promotion but Black men and white women are promoted. In the absence of any such example, one may be forgiven for thinking that the individuality of the grounds is indeed key to how discrimination is conceived, and that discrimination law is that which prohibits only the paradigmatic or central case of discrimination based on a single ground at a time.

This article seeks to test this inference. In particular, it seeks to understand the workings of the central case methodology in discrimination law theory, to test whether a prohibition on discrimination based on a combination of grounds (‘intersectional discrimination’) can be a legitimate case within the traditional ‘single-axis’ framework of discrimination law.

Three factors dictate the interest in this inquiry. First, theorists seem indifferent to discrimination based on several grounds such as discrimination against Black women based on both race and sex, making it seem as if discrimination law theory is solely about single-axis discrimination. This is despite the fact that the field has grown rapidly, especially in the last decade.^4^ General accounts of both *discrimination* and *discrimination law* have emerged.^5^ These accounts seldom go beyond what they consider the central/core/paradigmatic/focal cases of discrimination running along a single axis, and of discrimination law as prohibiting such single-axis discrimination, viz racism, sexism, ableism, homophobia, transphobia, ageism, etc.^6^ Where this pattern is exceeded, discussion quickly turns into examples (perhaps of non-central cases) based on veganism, smoking, rewarding one’s children, or being vain or a bully, but seldom towards discrimination based on multiple grounds.

Secondly, the indifference of theory appears complicit in the doctrinal resistance to intersectionality. ‘Combination discrimination’ based on two grounds in section 14 of the UK Equality Act 2010 remains unenforced to date. There is no explicit provision of such kind in the US Civil Rights Act 1964 or the Canadian Charter of Rights and Freedoms 1982. The minimal recognition that there has been remains at the level of appellate courts,^7^ with the US and Canadian Supreme Courts having dodged any chance of going beyond single-axis discrimination.^8^ The Indian Constitution addresses discrimination that is based ‘on grounds only of religion, race, caste, sex, place of birth or any of them’, interpreted as a prohibition on discrimination that is based *only* on a single ground.^9^ The European Union too addresses discrimination on individual grounds and has explicitly denied that discrimination could be based on a combination of them.^10^ South Africa seems to be the only prominent outlier in having recognised discrimination based on ‘one or more grounds’ both in the text of its constitution and in constitutional adjudication.^11^ Nonetheless, by and large, intersectionality remains marginal across jurisdictions.^12^

Thirdly, this neglect in discrimination law theory and practice alike continues despite the ‘burgeoning field of intersectionality studies’^13^ and the particular contribution discrimination lawyers have made to the field.^14^ ‘Intersectionality’, conceived specifically in the context of discrimination law, has had considerable traction in discrimination law scholarship.^15^ Yet that scholarship bears little on the general inquiries in the area. Theory seems methodologically committed to working with stripped down examples of discrimination in an apparent attempt to say something general and fundamental. The example of discrimination against Black women is perhaps considered too complex or specific to be engaged with for the purposes of theorising. But in the absence of such acknowledgement, let alone engagement, the discourse on intersectionality or intersectional discrimination seems to exist in a parallel universe of its own. This is despite it being a theoretical discourse in its own right, with a much longer history and bandwidth, having existed since the mid-19th century and having proliferated across a range of disciplines, including economics, sociology, anthropology, feminist studies, political science, history and literature.^16^

This article shows that none of these positions is actually licit. In particular, it argues that the indifference to complex (intersectional) forms of discrimination in the theory of discrimination law can be rectified. Intersectional discrimination may be more central to the theory of discrimination law than hitherto imagined. This means that the theory should not be construed as supporting the existing doctrinal dissonance with intersectionality, and that intersectionality theory *qua* central case methodology has tremendous potential to contribute to the general theory of discrimination law in terms of unravelling the nature of discrimination to be prohibited and the nature of legal norms prohibiting such discrimination.

Thus, the main argument is that while the prohibition of intersectional discrimination appears to be no more than a peripheral case of discrimination law, it should actually be seen as ‘more than’ a central case itself since it is epistemically more capable of capturing the relative disadvantage to be redressed through discrimination law. The argument is developed in three steps. Section 2 presents a brief account of the central case methodology and shows that it may be read as identifying not only central, analogous or peripheral cases, but also cases which are ‘more than’ central cases when they align with the moral point or purpose of an enterprise, per Lon Fuller. Section 3 shows that the examples from which discrimination law is theorised, without exception, are single-axis cases. Correspondingly, the key features of discrimination law identified from these examples prove to be an awkward fit for intersectional cases. Section 3 uses the theory of discrimination law offered by Tarunabh Khaitan and the four key features he identifies to test whether intersectional discrimination can be explained within those features. It concludes that the features, while relatable, do not directly or sufficiently explain the nature of intersectionality as a form of discrimination to be prohibited in discrimination law. More conceptual work needs to be done to fully understand a prohibition on intersectional discrimination as part of the general norms of discrimination law. This section shows what this work may entail, and the final section shows why this work is necessitated by the central case methodology. Section 4 argues that the central case methodology is not exhausted with identifying central cases or their generalisable features, but also concerns cases which truly befit its purpose. If the purpose of discrimination law is to reduce relative disadvantage, a norm prohibiting intersectional discrimination may be more than a central case of discrimination law, because it is capable of addressing not just the unique form of discrimination associated with a combination of grounds, but also discrimination based on individual grounds. It can thus address relative disadvantage more comprehensively than single-axis discrimination and can thus achieve the purpose of discrimination law fittingly. The article concludes that this reckoning is important for discrimination law theory, not only in appreciating the true significance of intersectionality, but also in applying the central case methodology correctly.

One thing bears clarification before launching further. This article is really about the central case methodology, in that: it provides a reading of the central case methodology generally; then specifically in discrimination law theory; and finally via intersectionality theory. Still, its focus is narrow. The article considers the ways in which the central case methodology is invoked in discrimination law, with the sole purpose of testing whether its invocation leaves space to accommodate intersectional discrimination or norms prohibiting discrimination based on several grounds at once. But the central case methodology can be wielded for many purposes.^17^ In the field of discrimination law, it has been popular in studying both the central case of ‘discrimination’^18^ and the central case of ‘discrimination law’.^19^ The two inquiries are distinct but closely linked in most accounts.^20^ Colm O’Cinneide describes the link elegantly:

the ‘central case’ of discrimination, [is] the core or paradigm form of discriminatory behaviour, whose moral ‘wrongness’ justifies legal intervention to protect potential victims and thus constitutes the primary rationale for the system of discrimination law taken as a whole.^21^

Seen this way, discrimination law is perhaps no different from other areas of law where the central case methodology is used to both understand the nature of moral wrongness of, say, a particular crime like murder and to justify how it is prosecuted and punished in criminal law.^22^ Similarly, this article is about the nature of discrimination—including intersectionality—as well as about the nature of discrimination law—including norms which go beyond the prohibition of single-axis discrimination. The article shows that the understanding of the structure of discrimination in terms of intersectionality theory (as in the grounds it is based on) helps understand the norms of discrimination law per se, thus treating the two inquiries as related. In fact, as it will go on to show, the methodological move to treat these inquiries as *too* distinct is itself problematic in that it ends up compromising the central case methodology, which is about both the examples invoked to theorise and the generalisable features identified from those examples to serve the point or purpose of an enterprise. In the absence of intersectional examples, we compromise understanding discrimination in its full complexity and intensity, and end up imagining discrimination law as removed from or unconcerned with such discrimination, thus straying from the purpose of discrimination law, which is to address relative disadvantage, broadly defined. To avoid this, the reader is invited to appreciate the two inquiries about the central case of discrimination *and* discrimination law in this context as fundamentally linked, because it will make a difference to the corrective of central case methodology being offered here. The hope is that this corrective will, in turn, orientate theoretical interventions in discrimination law towards intersectionality.

## 2. Central Case Methodology

The central case methodology involves identifying a case of a general category on the basis of the generalisable traits it possesses so that it helps understand the nature and other instances of that particular category of things. The purpose of the central case methodology is to stabilise the meaning of the category under consideration by giving it a prototype that provides a meaningful reference point for all inquiries. For example, Aristotle describes the central case of friendship as a relationship that is virtuous because of the mutual goodwill friends possess towards one another, for its own sake and because they are good characters themselves.^23^ This ‘perfect’ or ‘complete’ form of friendship helps understand the ‘true’ nature of friendship as much as it helps identify other instances of friendship which are not so. These other instances, albeit instances of so-called friendship, inasmuch as they too may be about goodwill towards one another and/or as possessed by good characters themselves, are not instances of the central case of friendship since they are ultimately for a different point or purpose (viz some benefit or pleasure).^24^

The central case methodology is most famously wielded in legal philosophy by John Finnis. He uses central cases or focal meanings as an analogical device to explain the nature of both central cases of law or legal systems (such as constitutional government) and peripheral or borderline cases (such as Hitler’s Germany).^25^ According to Finnis, ‘a term is analogical when its meaning shifts systematically (ie according to some principle or rationale) as one shifts from one context or use to another’.^26^ However, the point of the central case methodology is not really to sort out analogical from disanalogical cases, by insisting on traits which must necessarily be present in all things of a kind. The point, according to Finnis, is that:

Rather, one’s descriptive explanation of the central cases should be as conceptually rich and complex as is required to answer all appropriate questions about those central cases. And then one’s account of the other instances can trace the network of similarities and differences, the analogies and disanalogies, for example, of form, function, or content, between them and the central cases. In this way, one uncovers the ‘principle or rationale’ on which the general term (‘constitution’, ‘friend’, ‘law…) is extended from the central to the more or less borderline cases, from its focal to secondary meanings.^27^

The central case methodology is thus about studying in great detail the features of possible central, analogical or peripheral cases in terms of their form, function and content, and thus deriving the principle or rationale for calling them central, analogical or peripheral at all.

For Fuller, as for Finnis and Aristotle, the central case methodology is employed with reference to a moral aim or purpose. The purpose of understanding the nature of law and legal systems via the central case methodology is to understand not only that which *is* law, good or bad, but that which is *better at being law* because it aligns more truly with the moral purpose of law.^28^ As Maris Köpcke Tinturé explains in this context:

Properly understood, and in line with the classical (‘natural law’) tradition with which Fuller broadly associates himself, the law’s ‘purpose’ means a morally valuable end which law is uniquely capable of serving … Or, in other words, that the end is the means’ moral purpose.^29^

According to Fuller, such purpose is embodied in the Rule of Law or his eight principles of legality, which subject human conduct to the governance of rules. These principles further a range of values, such as autonomy, fairness, well-being, reciprocity, partnership and respect—values significant to human beings and hence significant to the operation of law itself. Law which is Rule of Law-compliant is in line with the purpose of serving values important to the well-being of human beings. It is thus not simply good law or a central case of law, but it is more than a central case itself, ‘more truly law’ for being so aligned with its purpose.^30^

## 3. Central Case of Discrimination Law

The central case methodology feeds into discrimination law theory in at least three ways. The starting point seems to be the selection of cases that purport to be cases of discrimination. Theorists sift through this selection to delineate the central case of discrimination as, say, segregation or apartheid, as opposed to excluding all Black friends from your birthday party or a city council promoting the Black history month, with the purpose of understanding what makes discrimination, such as in segregation or apartheid, *discrimination* as such. In line with the general central case methodology, the point here is not to deny some forms of discrimination as *discrimination*, but to understand the nature of discrimination that the field of discrimination law has been uniquely designed to address. Thus, secondly, theorising rests on the edifice of those cases which illuminate the features of both discrimination and the law prohibiting such discrimination, viz discrimination law. Finally, theories are tested against the ‘general justifying aim of discrimination law’,^31^ to see whether the structure of discrimination law so identified aligns with the overall point or purpose of discrimination law.

Intersectionality does not make a cut at any of these levels. Subsection A below shows that the examples of central cases brought up to theorise on the nature of both discrimination and discrimination law do not go beyond one ground at a time. The argument here is that this is insufficiently appreciative of the central case methodology in gleaning over complex and diverse examples of discrimination, if discrimination is so limited to one ground alone. Subsection B shows that the lack of diversity of case selection in excluding intersectional examples reflects in the theory derived from those examples, in that the features of the theory seem relevant but do not themselves suffice to explain intersectional discrimination fully.

### A. Case Selection

In 1976, in one of the earliest essays exploring the contours of discrimination law, Paul Brest identified ‘The heart of the antidiscrimination principle [as] its prohibitions of race-dependent decisions that disadvantage the members of the minority groups’.^32^ Brest agreed that although ‘the antidiscrimination principle can be and has been extended to encompass a variety of other traits, including alienage, illegitimacy, and sex … the inquiries necessary to evaluate such extensions [lay] beyond the scope’^33^ of his essay. The theorisation of discrimination law in the United States thus began with an almost exclusive focus on race discrimination defined as race-dependent decisions that selectively disadvantaged members of a minority group. In 1992, Larry Alexander, in what is often credited as a field-defining article, broadened the realm of inquiry across a range of characteristics, viz sex, race and religion.^34^ However, the inquiry was firmly rooted in *one* discrete cause of discrimination at a time. Alexander used anti-Semitism, prejudice against Blacks and bias against homosexuals as examples animating his philosophical journey, distinguishing in the process these central cases from seemingly non-central ones, such as discrimination in the choice of romantic partners based on race or beauty. Writing shortly after Alexander, Cass Sunstein’s exposition of discrimination law similarly devoted itself to distinctions between groups that were disadvantaged by *one* kind of discrimination. The work was rich in data giving ‘a sense of the world of discrimination’ and, in particular, information about ‘racial and gender disparities in the United States’.^35^ But racism and sexism were treated as hermetically sealed categories, with no information indicating that they combined and interacted at all. Sunstein’s data, and his central examples of racism and sexism, when they spoke of the disadvantage suffered by Blacks and women, were thus only really speaking of Black men and white women, leaving the position of Black women uninterrogated. Like Gardner in the UK, Brest, Alexander and Sunstein in the United States set the tone for the emerging theoretical interest in discrimination law that started to pervade both sides of the Atlantic.^36^ The moral character of discrimination was to be adjudged along the lines of examples that were precisely about *a* kind of discrimination; and it was such discrimination that norms of discrimination law were aimed at.

The absence of a broader set of, possibly intersectional, examples in theoretical accounts is surprising because of their contemporaneity with critical race theory. We know that Black women’s position was different and merited being considered on its own because this was exactly the time when this message, or the theorisation of Black women’s disadvantage in intersectionality theory and critical race feminism, was at its peak.^37^ Anti-essentialism^38^ and multiple consciousness^39^ as jurisprudential methods were being taken up in the academe. So, while discrimination law was only just beginning to theorise, Black feminism, both in theory and practice, had been vociferously interrogating the disadvantage faced by Black women since the mid-19th century.^40^ They had shown that Black women’s disadvantage was not subsumed by the generic categories of racism and sexism, which had come to be defined through the experiences of those who were disadvantaged exclusively by their race or sex. The category of sexism thus explained the position of women who were disadvantaged *only* as women and not as Blacks or Asians, or by their poverty, sexual orientation, age or disability.

In fact, in 1989, Kimberlé Crenshaw had pointedly argued that discrimination law as a field only benefited those singularly disadvantaged, leaving those who suffered from multiple sources of disadvantage at the bottom of the hierarchy. Her ‘basement’ imagery explained the structure of inequality or discrimination vividly:

Imagine a basement which contains all people who are disadvantaged on the basis of race, sex, class, sexual preference, age and/or physical ability. These people are stacked—feet standing on shoulders—with those on the bottom being disadvantaged by the full array of factors, up to the very top, where the heads of all those disadvantaged by a singular factor brush up against the ceiling. Their ceiling is actually the floor above which only those who are *not* disadvantaged in any way reside. In efforts to correct some aspects of domination, those above the ceiling admit from the basement only those who can say that “but for” the ceiling, they too would be in the upper room. A hatch is developed through which those placed immediately below can crawl. Yet this hatch is generally available only to those who—due to the singularity of their burden and their otherwise privileged position relative to those below—are in the position to crawl through.^41^

The ‘hatch’ Crenshaw describes is discrimination law. Her critique was that the hatch was defined and limited by ‘uncritical and disturbing acceptance of dominant ways of thinking about discrimination’.^42^ The ‘but for’ thinking in discrimination law had allowed only single-axis discrimination to be seen and addressed as discrimination at all. Intersectional discrimination based on a combination of grounds, such as race *and* sex, had no way to be accounted for on its own without being fragmented into separate categories of race discrimination and sex discrimination. It was thus excluded from the ‘established analytical structure’ of discrimination law.^43^ The surge in theorising in discrimination law that occurred after this critique, and long after the ‘the heady days of intersectional scholarship’,^44^ ie the 1970s and 1980s, did not, however, pick up on the critique.

This is true at least in respect of the case selection in subsequent scholarship theorising both on discrimination law and discrimination generally. Take, for example, Khaitan’s thought experiment to identify norms of discrimination law from eight possible scenarios.^45^ The scenarios involve people acting or omitting to do something on grounds of race, gender, weight, eye colour, employment status, disability, etc. In one scenario a landlord is prohibited from refusing accommodation to a person on the ground of his or her race, while in another scenario public employers are mandated to encourage women’s participation in workforce. None of the scenarios invoke a combination of grounds. Each scenario involves one ground. Nothing about the examples that Khaitan brings up as central (and non-central) cases is causally complex in terms of the multiplicity of identity characteristics or forms of disadvantage that purported norms of discrimination law attend to.

Along similar lines, Kasper Lippert-Rasmussen’s opening list of ‘paradigmatic’ and ‘non-paradigmatic’ cases of discrimination are all unidimensional. Predictably, for him, ‘paradigmatic cases [of discrimination] are the denial of women’s right to vote and the way in which non-white South Africans were treated under apartheid’.^46^ Non-paradigmatic cases of ‘differential treatment’ are also based on one ground at a time—whether one is a smoker or in need of a life-saving treatment. Single-axis discrimination seems to be the norm both for central and non-central cases.

Lippert-Rasmussen and Khaitan offer general and comprehensive accounts of discrimination and discrimination law, respectively. It is not that they think that their accounts do not apply to cases wider than those that they theorise from. In fact, they hope that they do.^47^ But there is something to be said for the examples they theorise from in conceiving the relationship between an act or omission and the grounds it is based on in order for it to constitute discrimination that is to be prohibited by norms of discrimination law. Examples of discrimination, from the very beginning, tend to be those based neatly on a single ground at a time. Intersectionality is largely ignorable at this stage of their theoretical quests.

Similarly, in Hugh Collins’s account of discrimination law, he proceeds with the understanding that ‘The central case of prohibited conduct is less favourable treatment of another person on grounds of (or because of) their race, sex, or *one* of the other protected group classifications’.^48^ Yet, he brings up the case of discrimination against white males, ostensibly to show that a seemingly non-central case of discrimination may equally end up being a case of discrimination; that discrimination *can* occur against those otherwise privileged by both their race and sex.^49^ However, nothing is said of discrimination experienced by those disadvantaged by both their race and sex, viz Black or Asian women. If at all, the stray reference to white males ends up privileging their seemingly non-central case of intersectional discrimination. In the scheme of Crenshaw’s basement metaphor, it appears to place those who are not otherwise disadvantaged at the top of the pecking order.

This trend persists even in philosophical inquiries on the nature of discrimination. In Hanoch Sheinman’s work, he draws up five ‘paradigmatic cases’ which we ‘all agree involve discrimination’.^50^ They are creative illustrations of what is wrong about differential treatment. In the first example, Sheinman rewards only his daughter and not his son when both his children finish their vegetables. The sense of unfairness is palpable to the son, who is later rewarded after protest. The next example concerns a Black offender who receives a harsher sentence from a judge than a white offender. In the third instance, a female candidate loses out to a male candidate for a job. In the fourth example, Sheinman refers to hate crimes—for example, when someone is beaten up because they are gay. Finally, Sheinman brings up the locus classicus of *Brown v Broad of Education*^51^ and the wrongness of segregation based on race. All of his paradigmatic cases are unidimensional. It appears that what we all agree on, for the purposes of theorising on discrimination or discrimination law, is limited to single-axis discrimination in a popular or traditional sense. But what is popular and traditional may hide what is more complex and worthy of attention.

The separation of young children from their families at the United States–Mexican border; the neglected incarceration of Black women as opposed to the oft-cited (and equally problematic) incarceration of Black men; sexual harassment of working women especially from a low socio-economic background; the phenomenon of corrective rape of lesbians in South Africa; and the continuing segregation in schools (in the United States, schools are more segregated now that at the time of *Brown*) that is no longer simply about ‘race’ but about poverty, class, disability and gender^52^ are just some additional examples of discrimination that do not attract theoretical attention. They are not traditional cases of single-axis discrimination, like racial segregation or unequal pay for women, but they seem, even at first blush, complex and morally urgent, like any other example of discrimination. They are all causally complex in that they seem to be based on more than one ground of discrimination. They deserve consideration because the central case methodology, per Finnis, is based on a rich and complex description of central and non-central cases for tracing the similarities and dissimilarities between them to the greatest possible extent. It is its comprehensiveness, and not a narrow focus on one type of case (in this case, single-axis discrimination), that marks the central case methodology. This level of detail is only possible, in turn, when our examples are rich and complex to begin with. This has not been the case with theory, which seems to only work with single-axis discrimination that is universally condemned (segregation and unequal pay) or is trivial (not rewarding children equally for eating vegetables).

This is not to say that theoretical accounts necessarily trade in truisms or trivia. That is certainly not the claim here. It is important to stress that we should resist calling one type of discrimination trivial *because* it is single axis. Rather, the very brief snapshot of the field presented above shows that it is because the examples of central cases have been of single-axis discrimination that theory seems to be steering clear of examples of causally complex forms of discrimination that are based on several grounds.^53^ And it is the latter set of examples that is neither obvious nor trivial, and hence purposeful for central case methodology as outlined in section 2. Theory just has not had much to say about such examples as central cases or otherwise.

### B. *Features of Discrimination Law*

Even though examples of central cases do not go beyond single-axis discrimination, it is possible that the general features of discrimination law they help identify extend to intersectional discrimination.^54^ That is, after all, what the central case methodology hopes to do. For example, Khaitan claims that his model of discrimination law applies generally, and beyond the central cases it is based on. In his words, ‘everything that lies outside the central case must either be justified as its logical extension or stand condemned as linguistically insupportable or possibly illegitimate’.^55^ It is therefore appropriate to test whether intersectional discrimination, which is positively outside the central cases Khaitan invokes, can be justified based on the general features of discrimination law he identifies or if it stands condemned as illegitimate. It is also particularly appropriate to focus on Khaitan here, given that he not only identifies some features, but tries to make sense of the whole field of discrimination law.^56^ His is thus a rare account of discrimination law whose purported features are aimed at extending to exactly the kind of cases being talked of here: intersectional discrimination.^57^

According to Khaitan, the *essence* of discrimination law is found in four conditions that run across all norms of discrimination law: the *personal grounds condition*, the *cognate groups condition*, the *relative disadvantage condition* and the *eccentric distribution condition*.^58^

The personal grounds condition requires some connection between the act or omission (that is prohibited or permitted by the norm) and the personal characteristics or attributes (‘grounds’) people possess. The term ‘grounds’ here is used in a technical sense in that not any ground of action (or omission) counts, but only those that connote ‘certain types of characteristics that persons have, such as race, sex, religion, weight, sexual orientation, age, disability, eye-colour, physical appearance, and marital status’.^59^ On this condition, it should be fairly straightforward to imagine a norm prohibiting intersectional discrimination, ie discrimination based on a combination of grounds, to be as much of a norm of discrimination law as a norm prohibiting single-axis discrimination. There is nothing in the personal grounds condition which a norm prohibiting intersectional discrimination cannot satisfy. All that it requires is *some connection* between the act or omission on the one hand and the grounds on the other. This connection is nowhere delimited by the number of grounds on which the connection is made. Even if an act or omission is based on a combination of grounds, the connection is established.

In fact, as Khaitan himself notes, his first condition is established ‘twice over’ when two grounds are involved.^60^ He does not develop this thought, however. It appears to be no more than a figure of speech, unintentionally indicating a quantitative rather than a qualitative reading of intersectional discrimination. Intersectional discrimination, though, is not a ‘sum of’ its parts.^61^ Instead, it is defined by the co-constituted nature of disadvantage when grounds combine such that the causal effect of individual grounds cannot be segregated and must be understood as a whole.^62^ This means that intersectionality forges a connection not only between the act or omission and the grounds, but also between the grounds themselves. An example is useful here. Take a case of discrimination like segregation, which can be understood either as a case of single-axis discrimination based on race, sex or religion, such as with Jim Crow laws, apartheid and single-sex or single-faith schools, or as a case of intersectional discrimination based on two or more grounds, such as race and sex. Norms prohibiting Black hairstyles such as dreads and cornrows,^63^ prohibition of pregnant pupils in schools or clubs^64^ and punitive discipline policies which are six times more likely to affect Black girls than Black boys^65^ are all examples of segregation which are intersectional in nature. They impact Black women specifically because of their race *and* sex. Though segregation affects Black men, it does *not* affect Black men when based on norms to do with, say, women’s reproductive agency. Similarly, though norms of grooming do impact women as such, stereotypes associated with certain styles of grooming are not attached to white women. In fact, white women’s adoption of ‘Afro-hairstyles’ has been deemed fashionable and not signalling promiscuity, a stereotype attached to Black women exclusively.^66^ Racism and sexism thus combine in complex ways which affect Black women’s segregation uniquely and not simply twice as much. Such discrimination can be understood as based on *some connection* between a norm mandating segregation on the one hand and grounds on the other, if that connection is understood to mean the connection *between* grounds in the way that they combine to yield the kind of discrimination suffered by groups like Black women. So, while the personal grounds condition explains the nature of discrimination in single-axis cases of segregation as in apartheid (as based on race), it needs to be substantially elaborated on further to explain how grounds combine to yield intersectional discrimination and what we mean when we say that discrimination such as in the case of segregation is based on a combination of grounds. In sum, the personal grounds condition merely underscores the connection of an act or omission *to* grounds, and while intersectional discrimination meets this criterion, intersectional discrimination requires the personal grounds condition to acknowledge and include that the connection *between* grounds be appreciated just as much.

Khaitan’s second condition is the cognate groups condition according to which a protected ground must be capable of classifying people into more than one class, loosely called ‘groups’. For example, sex as a ground is capable of classifying persons into women, men and transpersons. But norms of discrimination rarely explicitly operate at the level of ‘groups’; rather, they work with ‘grounds’. We can say that they must do so because, ultimately, legal norms of discrimination establish the connection that is necessary for discrimination to occur; and that it is this quality of discrimination or the norms which prohibit it (to be based on grounds) that makes discrimination as a field of law special.^67^ The cognate groups condition thus can be imagined as a subcondition within the personal grounds condition to explain that the connection between an act or omission and particular grounds is that the act or omission affects two or more groups differently.

Seen in this light, since intersectional discrimination occurs on the basis of multiple grounds, it ends up classifying intersectional claimants who experience such discrimination across several groups at once.^68^ For example, in the case of discrimination against Black women, the two grounds of their claim—race and sex—classify Black women as a group different from white men, Black men and white women. This is because Black women share their race with Black men and their sex with white women, but are also different from either of the groups because of their sex and race respectively. When combined, the two grounds of race and sex *are*, in Khaitan’s terms, ‘capable of classifying persons into more than one class of persons’. It is just that the classification is no longer exclusive so that members of one group (Black women) are never members of another group (Black men and white women). But comparing Black women to cognate groups like Black men (on the basis of sex) and white women (on the basis of race) only captures aspects of discrimination which Black women do not share with Black men and white women because of their sex and race respectively; nor does it capture the experiences of discrimination they share with them on the basis of their race and sex respectively. Just how Black men and white women serve as Black women’s ‘cognate’ groups does not become clear at the level of the second condition. Like the personal grounds condition though, the cognate groups condition can be expanded to recognise how different cognate groups relate to one another, especially in the case of intersectional discrimination, which inevitably involves several cognate groups at once. But even on its own, this condition should not be construed such that intersectional discrimination gets reduced to being based on a single hybrid ground in the sense that race and sex somehow become a super or special sub-ground—racesex, race-sex or race+sex—to limit the cognate group for Black women to the category of white men alone.^69^ This is simply not possible. Black women do not get detached from the groups they already belong to, of Blacks and women generally, divorced from categories of race and sex completely, and launched into a super ground or category of their own. The implications of this would otherwise be that Black women are somehow neither Black nor female and that only Black men are Blacks and white women are women. The implausibility of this scenario has been slam-dunked by Black feminists.^70^ So, at the very least, this reading of the second condition should be avoided lest it obliterates the point of intersectionality, which is about the *intersection* of grounds rather than single hybrid grounds. Again, intersectionality is qualitative: about what happens when two or more grounds combine to yield the experience of discrimination complained of. Since the second condition only requires grounds to be capable of classifying people into more than one cognate group, it should be construed as accommodating of intersectionality, and should, in turn, reflect intersectionality by recognising not only the possibility of classifying people into groups, but also the *relationships* between groups when persons belong to two or more groups at once. Like the first condition, the second condition relates to intersectionality, but needs to reflect more of it, and in fact can do so if construed with specific attention paid to the qualitative difference between single-axis and intersectional discrimination.

It is the third condition where these qualitative implications really come to bear. According to this condition, members of at least one group (viz Blacks) defined by a ground (race) must be significantly more likely to suffer abiding, pervasive and substantial disadvantage than members of at least one other cognate group (whites).^71^ For an intersectional group like Black women, again, one may think that their disadvantage can only be appreciated as against one another cognate group, viz white men, who share neither of their personal characteristics. And although groups like Black men and white women do not seem obvious cognate groups for Black women under the second condition, they can be rather helpful in establishing the qualitative impact of discrimination under the third condition. Black women can show relative disadvantage as compared to Black men and white women—such that Black men’s experience of sex or gender is dissimilar to Black women’s experience of sex discrimination despite their common race; and white women’s experience of race is different from the racism Black women suffer despite their common sex. This does not eliminate what they do share with Black men and white women either—their experiences of race discrimination and sex discrimination on the basis of race and sex respectively. The kernel of intersectionality is thus that their relative disadvantage appears both similar to and different from all three groups—white men, Black men and white women.

Such relative disadvantage, as trenchant accounts of Black feminism show, is debilitating. For example, in the United States, Black women’s labour remains cheap (cheaper than both Black men and white women), they remain more prone to sexual harassment at work than other women and their economic conditions remain worse than all their possible cognate groups (including white men, white women, Black men, Asian men, Asian women etc).^72^ Their disadvantage is *abiding* in that it is a relic of disadvantage suffered since slavery.^73^ Black women had toiled as much as Black men in the fields and mines as slaves, and toiled even more at homes both of their own as Black homemakers and of their white owners, where they were mistreated by their mistresses and sexually exploited by their masters. Black women were also subjugated by Black men; thus, sexual harassment and violence were common both within and outside home. They were also uniquely exploited as sites for reproducing the next generation of slaves. All of these forms of disadvantage (slavery, unequal pay, sexual harassment, sexual exploitation, violence etc) are *substantial*, weighty on their own and even more so when appreciated in totality as the Black women’s condition. The disadvantage is *pervasive*, given that it is not confined to a particular sphere but cuts across Black women’s private and public lives. The abiding, pervasive and substantial nature of relative disadvantage suffered by Black women is thus a debilitating confluence of disadvantage which is both similar to other groups who were disadvantaged by their race (Black men) and sex (white women) but also quite different from them, because ultimately they experience racism and sexism together and not individually. As embodied beings, their position of relative disadvantage can only be understood as a whole. The third condition appears capable of accommodating such complexity of relative disadvantage if we recognise such disadvantage *inter se* across groups (say, between the groups of men and women) and *intra se* within a disadvantaged group (say, within the group of women between white women and Black women). But it is only when we modify the condition in this way to relate to both these forms of relative disadvantage between and across groups and not just leave it at saying that the disadvantage is relative in comparison to ‘at least one other cognate group’ that this condition actually makes sense for intersectional discrimination.

Khaitan’s last condition is eccentric distribution, so that a duty-imposing norm must be designed to benefit some but not all members of a beneficiary group. This condition is about the design of discrimination norms, especially distinguishing them from other norms of welfare law or socio-economic rights which seek to distribute benefits equally, to all members of an intended beneficiary group. Khaitan is right to point out that norms of discrimination are often not about equal distribution within an intended beneficiary group, not even aspirationally, let alone as an intended consequence of the operation of discrimination norms. Only some members of a group really benefit from discrimination norms which merely equalise *access* to benefit of those norms rather than equalise the actual benefit itself. So conceived, norms prohibiting intersectional discrimination may satisfy this condition. Just like the prohibition of discrimination based on a single ground, a prohibition on discrimination based on a combination of grounds—say, in accessing education, employment or housing—only ensures that groups like Black women, disabled women or Muslim gays access education, employment or housing without any limiting effect of the personal characteristics they possess, two or more of which combine to potentially limit access to these basic goods. A bar on intersectional discrimination may also simply ensure *access* to a benefit for groups intersectionally disadvantaged, but may not ensure that they receive a benefit without more.^74^

On the whole, intersectional discrimination seems to largely relate to the central features of discrimination law outlined by Khaitan. At a general level, intersectionality under his model may be said to be (i) based on grounds (ii) which divide people into cognate groups that are (iii) defined by the relative disadvantage between them, and (iv) merely equalise access to redress such disadvantage. But more needs to be said about each of these conditions to capture intersectional discrimination fully as prohibiting discrimination based on a combination of grounds which divide people into several possible cognate groups, resulting in relative disadvantage that is both similar to and also different from other groups at the same time, and thus only understood as a whole. As this section has shown, the generality of Khaitan’s conditions allow for more work to be done in this regard. Thus, there seems to be some leeway in the ‘hatch’ or the ‘established analytical structure’^75^ of discrimination law in accommodating intersectional discrimination, ultimately attending to Crenshaw’s appeal for considering Black women’s discrimination *as it is*, ie in light of intersectionality, to ‘sufficiently address *the particular manner* in which Black women are subordinated’.^76^

So far, so good. But this is not the complete story. We know that the central case methodology is not only about identifying the rationale on the basis of which central cases (single-axis discrimination) can be extended to understand other cases (intersectional discrimination), but also, importantly, about serving the moral point or purpose to which the enterprise is oriented such that cases which befit the purpose of the enterprise may be considered ‘more than’ central cases or better at being central cases themselves. The next section completes this arc.

## 4. *More Than a Central Case of Discrimination Law*

The central case methodology is about studying central cases in as much detail and complexity as possible. Theories of discrimination law adopt the central case methodology to study central, analogical and peripheral cases in great detail to reveal the principle or rationale on which they are based. Applying the central case methodology to theorising in discrimination law should thus involve consideration of intersectional, as much as single-axis, cases, especially since, as was argued in the previous section, intersectional discrimination seems to be an analogical case of discrimination law even though it is largely peripheral to the central cases actually considered in the process of theorising. Yet, as this part argues, intersectionality is important because it shows that a norm prohibiting intersectional discrimination may not just be a central, analogical or peripheral case of discrimination law, but may be more than a central case itself. This part shows that intersectional discrimination is such a case because it befits the purpose of discrimination law—which is to reduce relative disadvantage—as it embodies relative disadvantage more comprehensively than single-axis discrimination.

To appreciate this, recall that intersectional discrimination based on a combination of grounds is both similar to forms of discrimination associated with individual grounds and different from them, because it also includes the unique combination in which those grounds intersect.^77^ This relationship is often depicted by a Venn diagram.^78^ For instance, when spheres of sex (A) and race (B) discrimination intersect, the resultant area of discrimination (A∩B) shows properties of both sex (A) and race (B) discrimination because the resultant area (A∩B) *is* composed of both forms of discrimination—(A) and (B). But it also shows unique properties of its own which are *not* represented in the non-intersecting areas of sex (A – A∩B) and race (B – A∩B) discrimination. The intersecting area (A∩B) is thus defined by a dynamic which is both similar to and different from sexism (A) and racism (B) as a whole, and hence unique in its structure of discrimination in comparison to where sexism and racism do not intersect.^79^ In other words, intersectional discrimination on the basis of sex and race is to be understood as: (i) sexism and (ii) racism (because it *is* made up of those spheres), and also (iii) a combination of the two (because the spheres *also* intersect). It is because of this complexity of intersectional discrimination as including (i), (ii) and (iii) rather than just (i) or (ii) (as would be the case for single-axis discrimination) that it is capable of explaining discrimination more comprehensively. For this reason, intersectional discrimination has epistemic priority in being able to explain not only its own unique constitutive character made up of (i), (ii) and (iii) together, but also, consequently, being able to represent its constitutive parts—(i) and (ii). Sara Bernstein illustrates this in the context of discrimination against Black women:

The intuitive idea is that in understanding black womanhood, we thereby understand blackness and womanhood. Being a black woman explains being black and being a woman; features of blackness and womanhood are at least partially explained by black womanhood. Intersectional explanations are more informative than explanations exclusively involving the individual identity constituents.^80^

As Bernstein too recognises, this initially appears to be counterintuitive.^81^ This is perhaps because intersectional discrimination has often been understood as either too different from or too similar to its constitutive parts of discrimination based on individual grounds.^82^ It is rarely understood, *qua* intersectionality theory, as having to do with both, since it is composed of individual forms of discrimination as well as their unique combination. But when understood this way, it becomes capable of explaining both its similarities with single-axis discrimination, and hence single-axis discrimination based on individual grounds, and its differences with single-axis discrimination, and hence intersectional discrimination based on a combination of grounds. In Bernstein’s words, intersectional discrimination thus becomes ‘more informative’^83^ or ‘more explanatorily powerful’^84^ than single-axis discrimination and, indeed, ‘central to understanding how various dimensions of race, gender, sexual orientation, disability status, and class interact to yield more complex forms of discrimination than those suffered by persons who fall under only one category’.^85^

To illustrate, discrimination against Muslim women in Europe shows aspects of Islamophobia, which is also experienced by Muslim men in general in relation to their appearance and stereotypes associated with them; sexism, which is experienced by women in general in relation to their gendered roles such as childcare and domestic work; and also a combination of the two in relation to headscarf discrimination, which affects Muslim women in particular.^86^ Or, for example, Dalit women in India face caste-based discrimination, such as untouchability; sex-based discrimination, manifested in lower rates of educational attainment and employment for women; and also unique forms of discrimination, such as are embodied in the practice of dedicating Dalit women as *Devdasis*, *Joginis* or *Muralis* to temples for sexual slavery and bondage.^87^ Similarly, while white women are paid less than white men in the United States, ie 77 cents for every white male dollar, Black women are paid 61 cents in comparison, which is not only lower than white women and men, but also lower than Black men, who get paid 70 cents a dollar.^88^ This means that Black women are paid 39% less than white men, 21% less than white women and 13% less than Black men. Because Black women are paid less than white men, just as Blacks and white women are, their unequal pay embodies forms of racism and sexism generally; and since they fare worse than other Blacks who are male and women who are white, they also embody the specific form of discrimination they face as Black women.^89^

These examples show the relative disadvantage between groups sharply and in terms of the complex relationships between and within the disadvantaged groups. This is important for the purposes of discrimination law, which is essentially concerned with understanding and addressing relative disadvantage between groups.^90^ Since intersectional discrimination is capable of representing relative disadvantage between groups more comprehensively than single-axis discrimination, prohibiting intersectional discrimination would serve the purpose of discrimination law rather well.

There is support for this position in Khaitan’s own account of the purpose of discrimination law.^91^ According to Khaitan, the ultimate point of discrimination law is to promote personal well-being.^92^ Well-being depends on access to certain basic goods (negative freedom, an adequate range of valuable opportunities and self-respect) because they enable people to live a good life. An important aspect of this account is that it is comparative or relative in two respects. As Khaitan explains:

[T]he freedom we are entitled to also depends on the freedom that others enjoy. In other words, our liberty-interest is relative, because [the] basic goods that constitute this interest have an essential connection with what others enjoy … Furthermore, the state’s duty to secure sufficient liberty to all of us has an inbuilt priority for those who are least free. Thus, this account of freedom is comparative in two significant respects—it aspires to guarantee basic goods which are relative in character, and for those who lack these goods the worst off have a prior claim to have their access enhanced.^93^

The point of discrimination law is thus both to promote well-being by reducing relative disadvantage between groups and to promote the well-being of those worse off than others. Discrimination law, as Khaitan posits, is comparative or relative in both these respects. In fact, discrimination law is of special moral significance *because* it treats relative disadvantage seriously in both these respects, since it affects people’s access to basic goods and hence their access to a good life.^94^ Addressing relative disadvantage thus becomes ‘a legitimate—nay pressing—moral concern’ for discrimination law.^95^

Intersectional discrimination, ontologically speaking, is often worse than single-axis discrimination. As seen in the examples recounted so far, discrimination against groups such as Black women is experientially different from discrimination suffered by groups such as white women and Black men. Thus, sexual harassment of Black women looks different from sexual harassment of white women because they face unwanted sexual advances in heavily racialised terms, where their bodies are singled out *as Black bodies* and they are excessively targeted with racial epithets and ethnic slurs.^96^ Sexual harassment is also often more invasive for Black women, with harassment routinely turning into sexual abuse and violence. Hypersexualised, exotic and promiscuous images of the ‘Black jezebel’ exacerbate the levels of sexual harassment against Black women, although they remain more likely to report, and less likely to be believed and ultimately to obtain redress, than white women.^97^ Intersectional discrimination against Black women in this case thus does end up being experienced both differently and in a worse way than individual forms of discrimination. This may not necessarily be the case in all cases of intersectional discrimination, as this is, after all, an empirical matter.^98^ However, when it is the case that intersectional discrimination renders people worse off than others, it may assume urgency or priority in the second of the two respects Khaitan describes.^99^

It is in the first of the two respects where intersectional discrimination really matters. Intersectionality, after all:

resists a race to the bottom in a kind of disadvantage contest where intersectional disadvantage is understood as worse or more important in a mathematical sense. The importance of intersectionality lies in the appreciation of qualitatively distinct explanations of same and different patterns of group disadvantage, rather than their quantitative rendition of sorts.^100^

Intersectional discrimination is qualitatively salient in terms of its dynamics of sameness and difference in the patterns of relative group disadvantage, and it is this that resonates with the purpose of discrimination law. If discrimination law is concerned with addressing relative disadvantage (howsoever defined), intersectional discrimination, epistemically speaking, addresses not just the unique form of disadvantage associated with the combination of individual grounds, but also the disadvantage associated with the individual grounds themselves. A norm prohibiting intersectional discrimination, then, addresses relative disadvantage in a wider sense than a norm prohibiting single-axis discrimination. For example, prohibiting intersectional discrimination in the case of Black women not only addresses discrimination unique to them, say in relation to their hair, but also addresses sexism in general in relation to grooming requirements for women at work, as well as racism in general in relation to Black people’s access to employment. Thus, barring employers from regulating how Black women wear their hair, whether in afros, cornrows or braids, uplifts Black women as much as it has the potential for uplifting white women and their choice of appearance, and Black men’s participation in the workforce. It is exactly this sense that Black women convey in slogans like ‘lifting as we climb’ or ‘when and where I enter, we all enter’.^101^ Intersectionality is also seen as ‘transformative’ exactly for this reason—that it has the capacity to transform not just discrete forms of discrimination, but the entire (basement) structure of discrimination from the bottom up.^102^ In turn, it unlocks the potential for discrimination law to serve its own purpose to really reduce the relative disadvantage between groups, and not only for those either worse off (Black women) or marginally better off (white women or Black men), but for several of these disadvantaged groups at once.

To sum up, appreciating intersectional discrimination as explanatorily or epistemically prior to single-axis discrimination in capturing relative disadvantage makes intersectional discrimination central to central case methodology in discrimination law.^103^ This places intersectionality, as Catherine MacKinnon puts it, at ‘the legal center’ of discrimination law, such that ‘discrimination against a person consigned to the bottom of two fused hierarchies [becomes] the first and foremost and most evident case in discrimination law, not an afterthought to it’.^104^ It is thus from this point or central case that we should begin to theorise on discrimination law.

## 5. Conclusion

This article has argued that although intersectional discrimination appears to be no more than a peripheral case in discrimination law, especially in the selection of central cases from which discrimination law is theorised, it may be analogised in reference to the features of discrimination law those cases help identify. But that is not enough, since it is actually more than a central case in that it is uniquely in accord with the moral point or purpose of discrimination law, which is to address relative disadvantage. This is because intersectional discrimination has epistemic priority in explaining relative disadvantage in discrimination law. There is one main implication of this for the purposes of central case methodology: that in the absence of treating intersectional discrimination as key to theorising on discrimination law, we may end up compromising the methodology used to theorise at all, which, in Finnis’s words, is meant to be ‘conceptually rich and complex’. Indifference to intersectionality ultimately compromises this detail and depth, especially in understanding the structure of complex cases of discrimination and ultimately having those cases reflect back into the norms in discrimination law. It would matter to discrimination law theory to get this right, or it would essentially mean getting the central case methodology wrong.

## Footnotes

1 John Gardner, ‘On the Ground of Her Sex(uality)’ (1998) 18 OJLS 167 (emphasis supplied). See also John Gardner, ‘Liberals and Unlawful Discrimination’ (1989) 9 OJLS 1; John Gardner, ‘Discrimination as Injustice’ (1996) 16 OJLS 353; John Gardner, ‘Discrimination: The Good, the Bad, and the Wrongful’ (2018) 118 Proceedings of the Aristotelian Society 55.

2 Gardner, ‘On the Ground of Her Sex(uality)’ (n 1) (emphasis supplied).

3 ibid 169 (emphasis supplied).

4 Although theorising had happened earlier too, especially in the context of the United States, see eg Owen Fiss, ‘The Fate of an Idea Whose Time Has Come: Anti-Discrimination Law in the Second Decade after *Brown v Board of Education*’ (1974) 41 U Chi L Rev 742; Paul Brest, ‘In Defense of the Antidiscrimination Principle’ (1976) 90 Harv L Rev 1; Richard Posner, ‘An Economic Analysis of Sex Discrimination Laws’ (1989) 56 U Chi L Rev 1311; Larry Alexander, ‘What Makes Wrongful Discrimination Wrong? Biases, Preferences, Stereotypes and Proxies’ (1992) 141 U Pa L Rev 149; Richard Epstein, *Forbidden Grounds: The Case against Employment Discrimination Laws* (Harvard UP 1992); Cass Sunstein, ‘The Anticaste Principle’ (1994) 92 Mich L Rev 2410.

5 See eg Deborah Hellman, *When Is Discrimination Wrong?* (Harvard UP 2008); Sophia Moreau, ‘What Is Discrimination?’ (2010) 38 Philosophy and Public Affairs 143; Deborah Hellman and Sophia Moreau (eds), *Philosophical Foundations of Discrimination Law* (OUP 2013); Joseph Fishkin, *Bottlenecks: A New Theory of Equal Opportunity* (OUP 2014); Kasper Lippert-Rasmussen, *Born Free and Equal? A Philosophical Inquiry into the Nature of Discrimination* (OUP 2014); Tarunabh Khaitan, *A Theory of Discrimination Law* (OUP 2015); Benjamin Eidelson, *Discrimination and Disrespect* (OUP 2015); Iyiola Solanke, *Discrimination as Stigma: A Theory of Anti-discrimination Law* (Hart Publishing 2016); Sophia Moreau, *Faces of Inequality: A Theory of Wrongful Discrimination* (OUP 2020).

6 Though some have hoped that their accounts apply more widely and to possibly more complex cases of discrimination. See esp Khaitan (n 5) 128–9, 137; Solanke, *Discrimination as Stigma* (n 5) ch 6.

7 See notable attempts in the United States, esp *Jefferies v Harris County Community Action Association* 615 F2d 1025 (5th Cir 1980); *Lam v University of Hawaii* 140 F 3d 1551 (9th Cir 1994); in Canada, see *Falknier v Ontario* [2002] OJ No 1771 (Ontario Court of Appeal); *Baylis-Flannery v DeWilde* 2003 HRTO 28; *Flamand v DGN Investments* 2005 HRTO 10.

8 The Canadian Supreme Court has recognised intersectional discrimination in principle, in passing. *Mossop v Canada (Attorney General)* [1993] 1 SCR 554, 582; *Withler v Canada* [2011] 1 SCR 396 [58].

9 See the reasoning of the Court in *Air India v Nargesh Meerza* AIR 1981 SC 1829. cf *Navtej Singh Johar v Union of India* (Writ Petition (Criminal) No 76 of 2016) (decided on 6 September 2018) [36] (Chandrachud J) (casting doubt on whether the reasoning in *Nargesh Meerza* is still viable).

10 Case C-443/15 *Parris v Trinity College Dublin* [2017] ICR 313 [80] (‘while discrimination may indeed be based on several of the grounds set out in Article 1 of Directive 2000/78, there is, however, no new category of discrimination resulting from the combination of more than one of those grounds, such as sexual orientation and age, that may be found to exist where discrimination on the basis of those grounds taken in isolation has not been established’).

11 See esp s 9(3) of the South African Constitution and its application in *Hassam v Jacobs* 2009 (5) SA 572; *Mahlangu v Minister of Labour and Others* [2020] ZACC 24.

12 For a discussion on comparative law in this area, see Shreya Atrey, *Intersectional Discrimination* (OUP 2019).

13 Sumi Cho, Kimberlé W Crenshaw and Leslie McCall, ‘Toward a Field of Intersectionality Studies: Theory, Applications, and Praxis’ (2013) 38 Signs 785.

14 Kimberlé W Crenshaw, ‘Demarginalizing the Intersection of Race and Sex: A Black Feminist Critique of Antidiscrimination Doctrine, Feminist Theory and Antiracist Politics’ (1989) U Chi Legal F 139; Sandra Fredman and Erika Szyszczak, ‘The Interaction of Race and Gender’ in Bob Hepple and Erika Szyszczak (eds), *Discrimination: The Limits of the Law* (Mansell 1992); Kathy Abrams, ‘Title VII and the Complex Female Subject’ (1993) 92 Mich L Rev 2479; Nitya Duclos, ‘Disappearing Women: Racial Minority Women in Human Rights Cases’ (1993) 6 Canadian Journal of Women and the Law 25; Nitya Iyer, ‘Categorical Denials: Equality Rights and the Shaping of Social Identity’ (1993) 19 Queen’s Law Journal 179; Emily Carasco, ‘A Case of Double Jeopardy: Race and Gender’ (1993) 6 Canadian Journal Women and Law 142; Mary Eaton, ‘At the Intersection of Gender and Sexual Orientation: Towards a Lesbian Jurisprudence’ (1994) 3 Southern California Review of Law and Women’s Studies 183; Sarah Hannett, ‘Equality at the Intersections: The Legislative and Judicial Failure to Tackle Multiple Discrimination’ (2003) 23 OJLS 65; Aileen McColgan, ‘Reconfiguring Discrimination Law’ [2007] PL 74; Joanne Conaghan, ‘Intersectionality and UK Equality Initiatives’ (2007) 23 SAJHR 317; Elsje Bonthuys ‘Institutional Openness and Resistance to Feminist Arguments: The Example of the South African Constitutional Court’ (2008) 20 Canadian Journal of Women and Law 1; Marius Pieterse, ‘Finding for the Applicant? Individual Equality Plaintiffs and Group-based Disadvantage’ (2008) 24 SAJHR 397; Iyiola Solanke, ‘Putting Race and Gender Together: A New Approach to Intersectionality’ (2009) 72 MLR 723; Sandra Fredman, *Discrimination Law* (OUP, 2nd edn 2011); Dagmar Schiek and Anna Lawson (eds), *European Union Non-discrimination Law and Intersectionality* (Ashgate 2011); Dagmar Schiek and Victoria Chege (eds), *European Union Non-discrimination Law: Comparative Perspectives on Multidimensional Equality Law* (Routledge Cavendish 2008); Kate Malleson, ‘Equality Law and the Protected Characteristics’ (2018) 81 MLR 598.

15 Crenshaw, ‘Demarginalizing’ (n 14).

16 For a genealogy of development of intersectionality, see Ange-Marie Hancock, *An Intellectual History of Intersectionality* (OUP 2016).

17 John Gardner, ‘Nearly Natural Law’ (2007) 52 Am J Juris 1, 14.

18 See eg Hellman (n 5); Moreau, ‘What Is Discrimination?’ (n 5); Eidelson (n 5).

19 See eg Oran Doyle, ‘Direct Discrimination, Indirect Discrimination and Autonomy’ (2007) 27 OJLS 537; Khaitan (n 5).

20 Sandra Fredman’s work is distinctive in this regard, see Fredman, *Discrimination Law* (n 14). See also Gardner, ‘On the Ground of Her Sex(uality)’ (n 1); Solanke, *Discrimination as Stigma* (n 5).

21 Colm O’Cinneide, ‘Justifying Discrimination Law’ (2016) 36 OJLS 909, 911.

22 I am grateful to Leah Trueblood for making this point.

23 Aristotle, *Nicomachean Ethics*, Book VIII (WD Ross tr) <http://classics.mit.edu/Aristotle/nicomachaen.8.viii.html> accessed 20 July 2020.

24 See the discussion in Neera K Badhwar and Russell E Jones, ‘Aristotle on the Love of Friends’ in Christopher Grau and Aaron Smuts (eds), *The Oxford Handbook of Philosophy of Love* (OUP 2017).

25 John Finnis, *Natural Law and Natural Rights* (Clarendon Press 1980) 11.

26 ibid 20.

27 ibid 11. See also Gregoire Webber, ‘Asking Why in the Study of Human Affairs’ (2015) 60 Am J Juris 51, 71 (‘The central case method, on this understanding, resists the idea that a general theory of law sets out to identify law’s “essential features” or “necessary and sufficient conditions” or what law “invariably does.” No part of the complexity of the human experience is to be denied attention or dismissed merely because it is imperfect. What is more, the central case is not to be misconstrued as “about ideal cases, still less about ideal worlds untroubled by wrongdoing, scarcity, misunderstanding, and fear”—it is to be understood as fully responsive to reasons that favor law, reasons articulated in the circumstances of the human experience, and not in ideal circumstances that wish away that human experience’) (references omitted).

28 Lon L Fuller, *The Morality of Law* (2nd edn, Yale UP 1969) esp ‘Reply to Critics’ 197–202.

29 Maris Köpcke Tinturé, ‘Concept and Purpose in Legal Theory: How to “Reclaim” Fuller’ (2013) 58 Am J Juris 75, 85 (references omitted).

30 Fuller (n 28) 200. See Tinturé (n 29) 88. See also Vittoria Villa, who makes a similar point about Finnis and Neil MacCormick that, for them, ‘some examples of human activities are “more central” examples than others, because they better exemplify the values towards which the enterprise is oriented’. Vittorio Villa, ‘Neil MacCormick’s Legal Positivism’ in Maksymilian Delmar and Zenon Bankowski (eds), *Law as Institutional Normative Order* (Ashgate 2009) 53–4. Similarly, according to Julie Dickson: ‘in order for a legal theorist to determine what is to count as law for the purposes of his inquiry, he must take a stance on what that point or purpose is and understand the central case of law in terms of that which successfully achieves it *via* specific techniques’. Julie Dickson, ‘Is Bad Law Still Law? Is Bad Law Really Law?’ in Maksymilian Delmar and Zenon Bankowski (eds), *Law as Institutional Normative Order* (Ashgate 2009) 162. cf Kristen Rundle, *Forms Liberate: Reclaiming the Jurisprudence of Lon L Fuller* (Hart Publishing 2012).

31 Khaitan (n 5) 91.

32 Brest (n 4) 2.

33 ibid 5.

34 Alexander (n 4) 151.

35 Sunstein (n 4) 2412.

36 Fiss (n 4); Posner (n 4); Epstein (n 4); Gardner, ‘Discrimination as Injustice’ (n 1); Nicola Lacey, ‘From Individual to Group’ in Bob Hepple and Erika Szyszczak (eds), *Discrimination: The Limits of the Law* (Mansell 1992); Denise G Réaume, ‘Discrimination and Dignity’ (2003) 63 La L Rev 1; Nicholas Bamforth, ‘Conceptions of Anti-Discrimination Law’ (2004) 24 OJLS 693.

37 See some of the key works especially from the 1970s: Linda JM LaRue, ‘Black Liberation and Women’s Lib’ (1970) 8 Transaction 59; Toni Cade (ed), *The Black Woman* (Signet Books 1970); Frances Beale, ‘Double Jeopardy: To Be Black and Female’ (1971) reprinted in (2008) 8 Meridians 166; Jean Cooper, ‘Women’s Liberation and the Black Woman’ (1971) 63 Journal of Home Economics 521; Inez Smith Reid, *“Together” Black Women* (Third Press 1972); Joyce A Ladneh, *Tomorrow’s Tomorrow: The Black Woman* (Anchor Books 1972); Mae C King, ‘The Politics of Sexual Stereotypes’ (1973) 4 Black Scholar 12; Geraldine Rickman, ‘A Natural Alliance: The New Role for Black Women’ (1974) 6 Civil Rights Digest 62; Pauli Murray, ‘The Liberation of Black Women’ in Jo Freeman (ed), *Women: A Feminist Perspective* (Mayfield Publishing 1975); Gloria Hull, Patricia Bell Scott and Barbara Smith (eds), *All the Women Are White, All the Blacks Are Men, but Some of Us Are Brave: Black Women’s Studies* (Feminist Press 1977); Diane K Lewis ‘A Response to Inequality: Black Women, Racism, and Sexism’ (1977) 3 Signs 339; Bonnie Thornton Dill, ‘The Dialectics of Black Womanhood’ (1979) 4 Signs 543.

38 Elizabeth Spelman, *Inessential Woman: Problems of Exclusion in Feminist Thought* (Women’s Press 1990); Angela P Harris, ‘Race and Essentialism in Feminist Legal Theory’ (1990) 42 Stan L Rev 581.

39 Deborah K King, ‘Multiple Jeopardy, Multiple Consciousness: The Context of a Black Feminist Ideology’ (1988) 14 Signs 42; Mari Matsuda, ‘When the First Quail Calls: Multiple Consciousness as Jurisprudential Method’ (1989) 11 Women’s Rights Law Reporter 7; Chela Sandoval, ‘US Third World Feminism: The Theory and Method Oppositional Consciousness in the Postmodern World’ (1991) 10 Genders 1.

40 See the long genealogy of development of intersectional thought in Hancock, *An Intellectual History of Intersectionality* (n 16).

41 Crenshaw, ‘Demarginalizing’ (n 14) 151–2.

42 ibid 138.

43 ibid 140.

44 Patricia Hill Collins, ‘Foreword’ in Bonnie Thornton Dill and Ruth Enid Zambrana (eds), *Emerging Intersections: Race, Class, and Gender in Theory, Policy, and Practice* (Rutgers UP 2008) ix.

45 Khaitan (n 5) 26.

46 Lippert-Rasmussen (n 5) 1–2.

47 See esp Khaitan (n 5) 128–9, 137.

48 Hugh Collins, ‘Discrimination, Equality and Social Inclusion’ (2003) 66 MLR 16 (emphasis supplied).

49 ibid 19.

50 Hanoch Sheinman, ‘Two Faces of Discrimination’ in Hellman and Moreau (n 5) 32.

51 347 US 483.

52 Sandra Fredman, ‘The Right to Education and Substantive Equality: An Intersectional Reading’ in Shreya Atrey and Peter Dunne, *Intersectionality and Human Rights Law* (Hart Publishing 2020); on the overlap between direct and indirect intersectional discrimination, see Atrey (n 12) 156–62.

53 For an explanation of causal complexity in cases of intersectional discrimination, see Atrey (n 12) 81–5. See also Ange-Marie Hancock, ‘When Multiplication Doesn’t Equal Quick Addition: Examining Intersectionality as a Research Paradigm’ (2007) 5 Perspectives on Politics 63.

54 Missing here are the steps theorists take to work through the central cases in identifying the key features of discrimination law which make up its analytical structure. That is, we are skipping the process of extrapolation of features from single-axis cases and cutting straight to whether those features can accommodate intersectional discrimination. The process in the middle may, of course, be of value in learning about central case methodology more widely, but in the interests of space, it will have to be earmarked for another outing which is not delimited by intersectional aims.

55 Khaitan (n 5) 5–6.

56 O’Cinneide’s review of Khaitan’s work is useful to note: ‘in general, remarkably little commentary has been produced which focuses on more “macro” issues relating to the overall purpose, function and justification of discrimination law. The theoretical writing that does exist on this subject is often rich and insightful, but it also tends to be fractured, taking the form of scattered conversations rather than a shared common dialogue. Furthermore, much of this commentary remains partial or underdeveloped—and in particular struggles to keep pace with the fluid, dynamic and ever-expanding role played by discrimination law in contemporary society. Khaitan’s book sets out to fill this gap. It aims to provide a comprehensive theoretical account of the content and justification of discrimination law, which faithfully reflects how it has evolved over the last few decades.’ O’Cinneide (n 21) 910.

57 Khaitan (n 5) 69, 137, 152, 162–3 (giving explicit examples of Indian women and Dalit women). Solanke too offers her theoretical account of discrimination law to attend to intersectionality specifically. However, her focus is more limited and, as she describes, on ‘a more fundamental question to be answered: how do we decide which categories are to be regarded as—to use the American term—“suspect”? When should we worry about the use of personal attributes, characteristics or conditions as ratio? And more to the point for this book, when should legal protection be introduced to prevent and protect against this?’ Her theoretical inquiry, though geared towards intersectional discrimination, is still concentrated on the selection or logic of categories of discrimination, and, as she clarifies, in contrast with Khaitan, not about understanding the purpose of discrimination law but about answering the practical question of who should be protected in law. Solanke, *Discrimination as Stigma* (n 5) 3–4.

58 These do not exhaust the features of discrimination law for Khaitan. His theory is rich and comprehensive in its consideration of other features, including difference/commonalities between direct and indirect discrimination, choice of and basis of grounds, affirmative action duties etc. But for the purposes of the central case methodology, we can concentrate here on the very architectural features that are derived from the examples of central (and non-central) cases.

59 ibid 29.

60 ibid 163.

61 Crenshaw, ‘Demarginalizing’ (n 14) 140.

62 Rita Kaur Dhamoon, ‘Considerations on Mainstreaming Intersectionality’ (2011) 64 Political Research Quarterly 230.

63 See *Rogers v American Airlines* (1981) 527 F Supp 229 (SDNY); Paulette M Caldwell, ‘A Hair Piece: Perspectives on the Intersection of Race and Gender’ (1991) Duke LJ 365.

64 *Chambers v Omaha Girls Club* (1986) 629 F Supp 925; Regina Austin, ‘Sapphire Bound’ (1989) Wis L Rev 539.

65 ‘Brown Center Report on American Education: Race and School Suspensions’ (2017) <www.brookings.edu/research/2017-brown-center-report-part-iii-race-and-school-suspensions/> accessed 20 April 2020.

66 *Rogers* (n 63).

67 This may be true for indirect discrimination as much as direct discrimination, since indirect discrimination is, for example, under s 19 of the Equality Act 2010 (UK) defined ‘in relation to a relevant protected characteristic’ (s 19(1)), then proven in relation to groups which possesses that protected characteristic and those who do not (s 19(2)).

68 In fact, this rendering of the first two conditions shows that there is never *one* cognate group for an intersectional claimant to be compared with. In the real world, if we agree that multiple protected grounds exist, then surely multiple groups based on those grounds exist too. Thus, the ground of sex does not simply divide persons into men and women. In the real world, so long as the protected ground of race exists alongside sex, men and women are further classified into Black women, Black men, white women, white men, Latinas, Latinos, Asian women, Asian men and so on. Disability, age, sexual orientation, religion etc add further layers of complexity to human identity. This result is often attributed to intersectionality, as if the theory itself imposes such a level of complexity, adding too many grounds in the mix that divide people into ever smaller categories or groups. But this is simply the function of grounds: to divide people into groups, where members of one group are bound to be members of very many other groups creating a network of similarities and differences between the groups. In other words, it is reality that is intersectional; intersectionality is not being superimposed on discrimination law.

69 A fear first expressed in the notable failure of intersectionality in *DeGraffenreid v General Motors* (1976) 413 F Supp 142, 143 (United States District Court, Eastern District of Missouri) (‘The plaintiffs allege that they are suing on behalf of black women, and that therefore this lawsuit attempts to combine two causes of action into a new special sub-category, namely, a combination of racial and sex-based discrimination. The Court notes that plaintiffs have failed to cite any decisions which have stated that black women are a special class to be protected from discrimination. The Court’s own research has failed to disclose such a decision. The plaintiffs are clearly entitled to a remedy if they have been discriminated against. However, they should not be allowed to combine statutory remedies to create a new “super-remedy” which would give them relief beyond what the drafters of the relevant statutes intended’).

70 This is aptly captured in the title of a canonical text by Hull, Bell Scott and Smith (n 37): *All the Women Are White, All the Blacks Are Men, But Some of Us Are Brave*. See also Crenshaw’s response to the cases of *Pyane v Traveno* 673 F 2d 798 (5th Cir 1982) and *Moore v Hughes Helicopters, Inc* 708 F 2d 475 (9th Cir 1983) in Crenshaw, ‘Demarginalizing’ (n 14) 143–8.

71 Khaitan (n 5) 31.

72 See eg the snapshot provided in the Report of the Institute for Women’s Policy Research, ‘The Status of Black Women in the United States’ <www.domesticworkers.org/sites/default/files/SOBW_report2017_compressed.pdf> accessed 20 April 2020.

73 For these accounts, see bell hooks, *Ain’t I a Woman: Black Women and Feminism* (South End Press 1981); Angela Y Davis, *Women, Race and Class* (Vintage 1981).

74 A critique of the limitations of this condition, as there may well be, is beyond the scope of this article. It may be that discrimination law need not be so limited, and in fact is not so limited, say, as imagined in the affirmative action provisions in the Indian constitution and in the South African constitution.

75 Crenshaw, ‘Demarginalizing’ (n 14) 140.

76 ibid (emphasis supplied).

77 In the words of Deborah King: ‘Black women have long recognized the special circumstances of our lives in the United States: the commonalities that we share with all women, as well as the bonds that connect us to the men of our race. We have also realized that the interactive oppressions that circumscribe our lives provide a distinctive context for black womanhood. Near the end of the 19th century, Anna Julia Cooper, who was born a slave and later became an educator and earned a PhD, often spoke and wrote of the double enslavement of black women and of our being “confronted by both a woman question and a race problem.” In 1904, Mary Church Terrell, the first president of the National Association of Colored Women, wrote, “Not only are colored women … handicapped on account of their sex, but they are almost everywhere baffled and mocked because of their race. Not only because they are women, but because they are colored women.”’ King (n 39) 42 (references omitted). See also Sojourner Truth, ‘Woman’s Rights’ in Beverly Guy-Sheftall (ed), *Words of Fire: An Anthology of African-American Feminist Thought* (New Press 1995) 36; Anna Julia Cooper, *A Voice from the South* (OUP 1988) 31; Barbara Smith (ed), *Home Girls: A Black Feminist Anthology* (RUP 2000) xxxiv; Cho, Crenshaw and McCall (n 13) 787; Vivian M May, *Pursuing Intersectionality: Unsettling Dominant Imaginaries* (Routledge 2015) 37, 70–1; Atrey (n 12) 40.

78 This, though, is not without criticism, since it does give the impression that forms of discrimination such as sexism and racism exist in a pure state when not combined, rather than giving the impression that is considered more accurate in intersectionality studies—that there are no pure categories as such and the reality is always such that discrimination (or even identities) exists in complex, multifaceted ways. For now, though, the Venn diagram imagery should suffice in honing in on the dynamic of sameness and difference which defines intersectionality. See Mitu Gulati and Devon W Carbado, ‘The Fifth Black Woman’ (2001) 11 Journal of Contemporary Legal Issues 701, 705–6.

79 Catharine A MacKinnon, ‘Intersectionality as Method: A Note’ (2013) 38 Signs 1019, 1020 (‘Intersectionality fills out the Venn diagrams at points of overlap where convergence has been neglected, training its sights where vectors of inequality intersect at crossroads that have previously been at best sped through. This reveals women of color at the center of overlapping systems of subordination in a way that moves them from the margins of single-axis politics that has often set priorities for opposing inequality as if they did not exist. On this level, it addresses “the combined effects of practices which discriminate on the basis of race, and on the basis of sex”. As a categorical corrective, intersectionality, as is well known, adds the specificity of sex and gender to race and ethnicity, and racial and ethnic specificity to sex and gender … Categories that had been uniform and few become modulated and variegated as well as many’) (references omitted).

80 Sara Bernstein, ‘The Metaphysics of Intersectionality’ (2020) 177 Philosophical Studies 321, 331.

81 ibid 332.

82 Crenshaw, ‘Demarginalizing’ (n 14) 150 (‘Unable to grasp the importance of Black women’s intersectional experiences, not only courts, but feminist and civil rights thinkers as well have treated Black women in ways that deny both the unique compoundedness of their situation and the centrality of their experiences to the larger classes of women and Blacks. Black women are regarded either as too much like women or Blacks and the compounded nature of their experience is absorbed into the collective experiences of either group or as too different, in which case Black women's Blackness or femaleness sometimes has placed their needs and perspectives at the margin of the feminist and Black liberationist agendas’).

83 Bernstein (n 80) 331.

84 ibid 330.

85 ibid 321.

86 *Sonia Yaker v France*, Human Rights Committee, Communication No 2747/2016, CCPR/C/123/D/2747/2016; cf *SAS v France* [2014] ECHR 695. See also Suriyah Bi, ‘More than “Multiple Jeopardy”: Navigating the Legal System as a British-Muslim-Woman-Litigant-in-Person’ (2019) 8 Journal of Muslims in Europe 247, 263.

87 The practice is formally banned, but continues nevertheless. ‘Devdasi System Continues to Exist Despite Ban’ *Outlook* (29 May 2016) <www.outlookindia.com/newswire/story/devadasi-system-continues-to-exist-despite-ban-book/941639> accessed 20 July 2020.

88 According to data released in 2018 by the Economic Policy Institute, United States Census Bureau <www.census.gov/newsroom/press-releases/2018/income-poverty.html> accessed 20 April 2020. See also Ariane Hegewisch, ‘The Gender Wage Gap: 2017; Earnings Differences by Gender, Race, and Ethnicity’ (Institute for Women’s Policy Research 2018) <https://iwpr.org/publications/gender-wage-gap-2017/> accessed 20 April 2020.

89 A point also explained by Crenshaw in reference to the three cases, *DeGraffenreid v General Motors* (1976) 413 F Supp 142 (United States District Court, Eastern District of Missouri), *Pyane v Travenol* 673 F 2d 798 (5th Cir 1982) and *Moore v Hughes Helicopters, Inc* 708 F 2d 475 (9th Cir 1983): Crenshaw, ‘Demarginalizing’ (n 14) 148 (‘In *DeGraffenreid*, the court refused to recognize the possibility of compound discrimination against Black women and analyzed their claim using the employment of white women as the historical base. As a consequence, the employment experiences of white women obscured the distinct discrimination that Black women experienced. Conversely, in *Moore* … the court would not entertain the notion that discrimination experienced by Black women is indeed sex discrimination-provable through disparate impact statistics on women. Finally, courts, such as the one in *Travenol*, have held that Black women cannot represent an entire class of Blacks due to presumed class conflicts in cases where sex additionally disadvantaged Black women’).

90 The assumption here is that the point or purpose of discrimination law is, broadly, to address relative disadvantage, defined in some form, whether distributively, in terms of stigma, stereotyping and prejudices, as about dignity or lack of substantive freedoms between groups, or as a combination of these. I will keep the substantive differences between these accounts at bay for now since there is relatively little controversy that discrimination law considers the difference or gap between groups relevant, howsoever one may choose to define that difference or gap. See Chris McCrudden (ed), *Anti-discrimination Law* (2nd edn, Ashgate 2004); Bob Hepple, ‘The Aims of Equality Law’ (2012) 61 CLP 1; Colm O’Cinneide, ‘The Uncertain Foundations of Contemporary Anti-discrimination Law’ (2011) 11 International Journal of Discrimination and the Law 7.

91 His is, though, but one expression of the moral point or purpose of discrimination law. Other accounts based explicitly on equality, dignity, the absence of stereotyping or the promotion of social inclusion can also be tested for support of this position. Again, the emphasis here is on addressing *relative* or *comparative* disadvantage as the ultimate moral point or purpose of discrimination law, with such relative ‘disadvantage’ being howsoever defined substantively in terms of its moral wrongness.

92 Khaitan (n 5) 91 (‘the general justifying aim—the point or purpose, if you may—of discrimination is to secure an aspect of the wellbeing of persons by reducing the abiding, pervasive, and substantial relative disadvantage faced by members of protected groups. Such disadvantage … prevents a person from securely accessing three basic goods necessary to live a good life. These goods are negative freedom, an adequate range of valuable opportunities, and self-respect’).

93 ibid 113–14.

94 ibid 122.

95 ibid.

96 Andrea L Dennis, ‘Because I Am Black, Because I Am Woman: Remedying the Sexual Harassment Experience of Black Women’ (1996) Ann Surv Am L 555. See also Kimberlé Crenshaw, ‘Race, Gender, and Sexual Harassment’ (1992) 65 S Cal L Rev 1467; Kimberlé Crenshaw, ‘Mapping the Margins: Intersectionality, Identity Politics, and Violence against Women of Color’ (1991) 43 Stan L Rev 1241.

97 Dan Cassino and Yasemin Besen‐Cassino, ‘Race, Threat and Workplace Sexual Harassment: The Dynamics of Harassment in the United States, 1997–2016’ (2019) 26 Gender, Work and Organization 1121.

98 Atrey (n 12) 200–1 (‘Intersectionality has no quantifiable stakes. It is not as if intersectional discrimination is necessarily worse or more problematic than other categories of discrimination or that discrimination on two grounds is actually double discrimination and one on three grounds is triply wrong … This is because the multiplicity of grounds in an intersectional claim does not explain what is wrong about intersectional discrimination per se. Intersectional wrongs matter in a qualitative sense … [For example] [t]he case for higher indemnification should thus be made on a case by case basis based on the severity of disadvantage suffered by the claimant’).

99 For a similar prioritarian account, see Lippert-Rasmussen (n 5) ch 6, esp 165–70.

100 Atrey (n 12) 44.

101 Crenshaw, ‘Demarginalizing’ (n 14) 167. For an all-inclusive vision of intersectionality, see Austin (n 64); Chandra Talpade Mohanty, Ann Russo and Lourdes Torres (eds), *Third World Women and the Politics of Feminism* (Indiana UP 1991); King, ‘Multiple Jeopardy (n 39); Smith (n 77).

102 Atrey (n 12) 52–3.

103 I will have to leave it for another occasion to see what treating intersectional discrimination as a central or more than central case does to discrimination law theory, ie what features of discrimination law actually look like when imagined afresh from the reference point of intersectionality. This article has laid the groundwork for this inquiry by showing that: (i) such an approach to theorising is necessary if theorists wish to stay true to the central case methodology they purport to adopt; and (ii) how the features of discrimination law could be reimagined to accommodate intersectionality.

104 MacKinnon (n 79) 1028.

